# A 3-dimensional model for bronchial and arterial sleeve resection

**DOI:** 10.1016/j.xjtc.2024.10.012

**Published:** 2024-10-26

**Authors:** Yoshifumi Hirata, Kohei Hashimoto, Keisei Tachibana, Ryota Tanaka, Haruhiko Kondo

**Affiliations:** Division of Thoracic Surgery, Kyorin University, Tokyo, Japan

**Figure:**
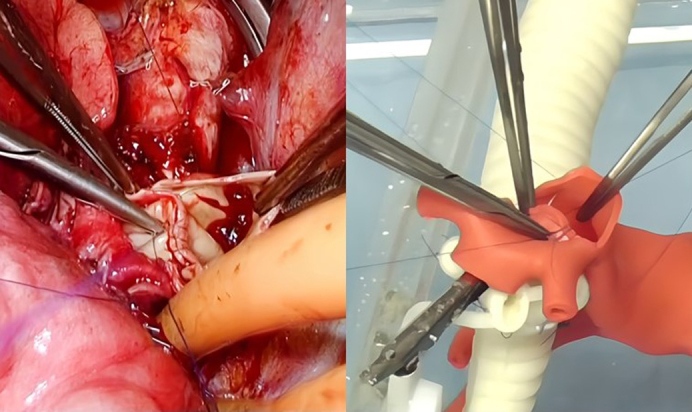
Comparison of pulmonary artery anastomosis between actual clinical case and on 3D model.

**Central Message**

A pulmonary artery model with precise human anatomy and texture was created in addition to our previously developed airway model. This armamentarium allows training of double-sleeve reconstruction.

Bronchial and pulmonary arterial double-sleeve lobectomy is a viable treatment option for centrally located lung cancer while avoiding pneumonectomy. Several studies show the short- and long-term benefits of avoiding pneumonectomy,^1^ highlighting the importance of this technique. However, because of its complexity and rarity, it is not easy for trainees to learn. Nevertheless, as all thoracic surgeons could encounter such a situation (even unexpectedly), thoracic trainees should be proficient in this technique. We have previously reported on a 3-dimensional (3D) bronchial model to supplement clinical training and successfully created a patient-specific model with a target invasive lesion.^2^ We have also demonstrated that training methods using this model improve the skills of thoracic trainees.^3^ The purpose of this study was to create a 3D model of the pulmonary artery as a training model for double-sleeve lobectomy.

## Methods

### Creation of the Model

The bronchial model described in the article is patented in Japan (JP7280446B). The pulmonary artery was extracted from the Digital Imaging and Communications in Medicine data of a 1-mm slice thickness of noncontrast computed tomography scan of the chest of a healthy volunteer and converted to 3D data. The data were converted to Standard Triangle Language format for 3D printing. Rigid plastic models were 3D printed and used as a framework to create the silicone mold. Soft urethane material was poured into the mold to create a pulmonary artery model with a 1.5 mm thickness (CrossMedical).

### Evaluation of the Model

The 3D model was evaluated by 4 board-certified thoracic surgeons experienced in double-sleeve resection (surgical exposure, rigidity, elasticity, and resistance to needles) with a 5-point Likert scale. A right upper double-sleeve lobectomy from an actual clinical case was performed on the model by the same surgeon for comparison. This study was approved by the institutional ethical review board (no. R05-020) May 17, 2023, and consent was waived because of its retrospective nature.

## Results

### Evaluation of the Model

**Figure 1:**
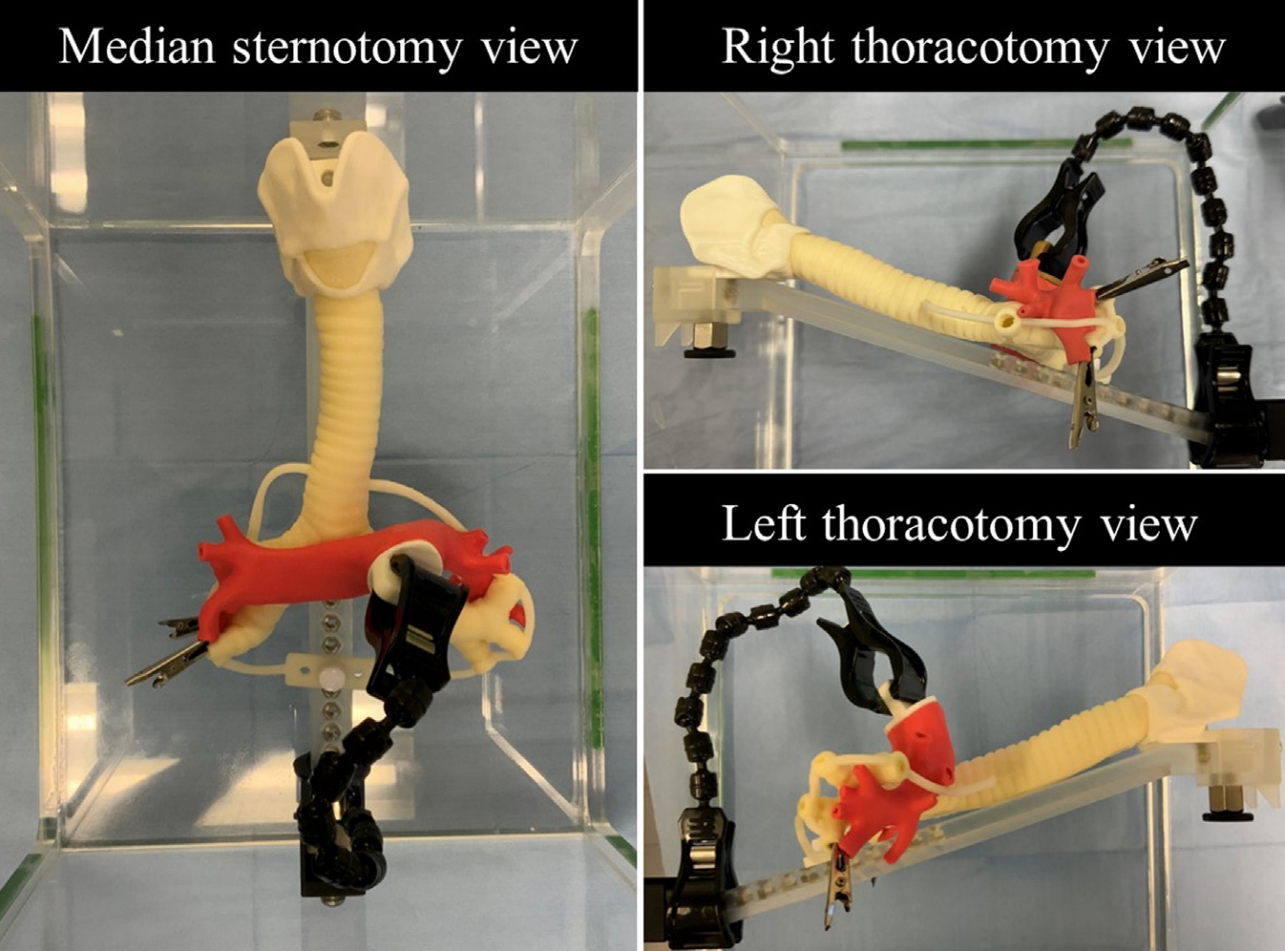
A 3-dimensional pulmonary artery model module is attached to the airway model. Pulmonary artery branches are clipped to the distal bronchus to prevent the distal portion from falling after resection.

A life-size 3D model of the pulmonary artery model was successfully created. It is accessible from a median sternotomy view, a right thoracotomy view, and a left thoracotomy view (Figure 1). Evaluation of the pulmonary artery model by the 4 thoracic surgeons was generally acceptable (median score [range] on a 5-point Likert scale: surgical exposure 4.5 [3-5], rigidity 3.0 [2-5], elasticity 4.0 [4-4], resistance to needles 4.0 [2-4], and resistance to tying 4.0 [4-5]).

### Clinical Case and Comparison With Simulation Surgery

**Figure 2:**
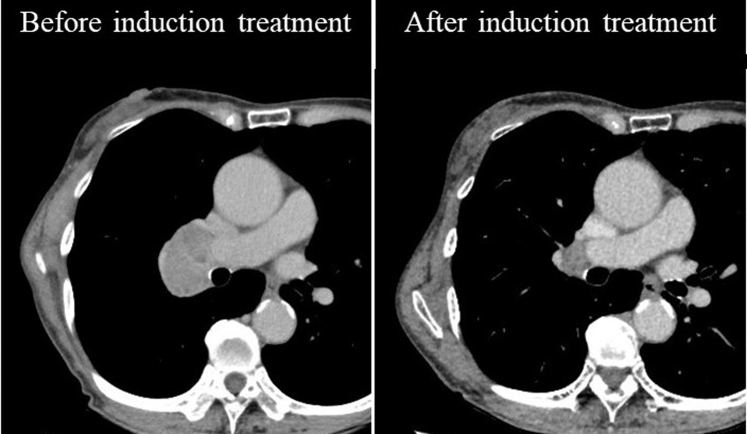
The metastatic 11R lymph node was remarkably enlarged, which seemed infiltrating the pulmonary artery and bronchus. After induction chemoradiotherapy, the lymph node was significantly reduced in size.

The clinical case was a 77-year-old female patient with right upper lobe pulmonary squamous cell carcinoma and metastatic 11R with extranodal invasion (cT1bN1M0 and ycT1aN1M0) who underwent a right upper lobe double-sleeve resection and reconstruction after induction chemoradiotherapy (Figure 2). This double-sleeve resection and reconstruction procedure was successfully reproduced on the 3D model (Video 1).

## Discussion

In this study, we successfully created a 3D pulmonary artery model, in combination with a previously developed airway model, that can simulate double-sleeve resection. To the best of our knowledge, our model is the first real-size operable pulmonary artery model reported in the literature.

Recently, simulation using animals (live or extracted organs) and cadavers became popular because of the increasing demand for off-the-job training of surgical trainees. However, there are many challenges in these training methods such as cost, limited access, anatomical differences, and ethical controversies regarding experimental animals. Surgical simulation using 3D models that accurately mimic human anatomy has recently been reported in neurosurgery and orthopedics^4^ as well as our model in thoracic surgery.^2^ Training with the models is not limited by location or time, allowing for repetitive practice. This may increase the learning efficiency of this complex procedure. By using a patient-specific model that includes invasive lesions, it may be possible to provide more precise simulations than cadaver surgical training. With increasing reports of sleeve resection in thoracoscopic surgery, we are also developing a chest wall module for simulation in video-assisted and robotic approaches.

Recently, the safety of sleeve resection after preoperative treatment with chemoimmunotherapy for locally advanced non−small cell lung cancer has been reported.^5^ The technical demands of reconstructive surgery may increase with the further evolution of perioperative treatment strategies. Therefore, even if the reconstructive procedure during pulmonary resection is not frequent, it is imperative to for the current and next-generation surgeons to be prepared and training with our models may have roles in this context.

This study is limited by the small number of trials, the inability of blood leakage tests, and the inability of tension reducing or dissection techniques. Because this model was the first of its kind, the scoring used to evaluate our 3D model was not fully validated; users should be aware of these aspects when using our simulation in the clinical context. A prospective multicenter study is warranted to further validate the effectiveness of training with the 3D model.

## Conclusions

A 3D pulmonary artery model was successfully created in addition to the airway model, and the double-sleeve reconstruction procedure was reproduced on the model.

## Conflict of Interest Statement

The authors reported no conflicts of interest.

The *Journal* policy requires editors and reviewers to disclose conflicts of interest and to decline handling or reviewing manuscripts for which they may have a conflict of interest. The editors and reviewers of this article have no conflicts of interest.

## Supplementary Data

Video 1 Comparison of the right upper double-sleeve resection on the actual patient and on the model performed by the same surgeon. Video available at: https://www.jtcvs.org/article/S2666-2507(24)00446-2/fulltext.
