# Supplementary figures and images for: A 3-dimensional model for bronchial and arterial sleeve resection
Source: JTCVS Tech. 2024 Oct 26;29:183–5. doi: 10.1016/j.xjtc.2024.10.012 (PMC11845382; doi:10.1016/j.xjtc.2024.10.012)

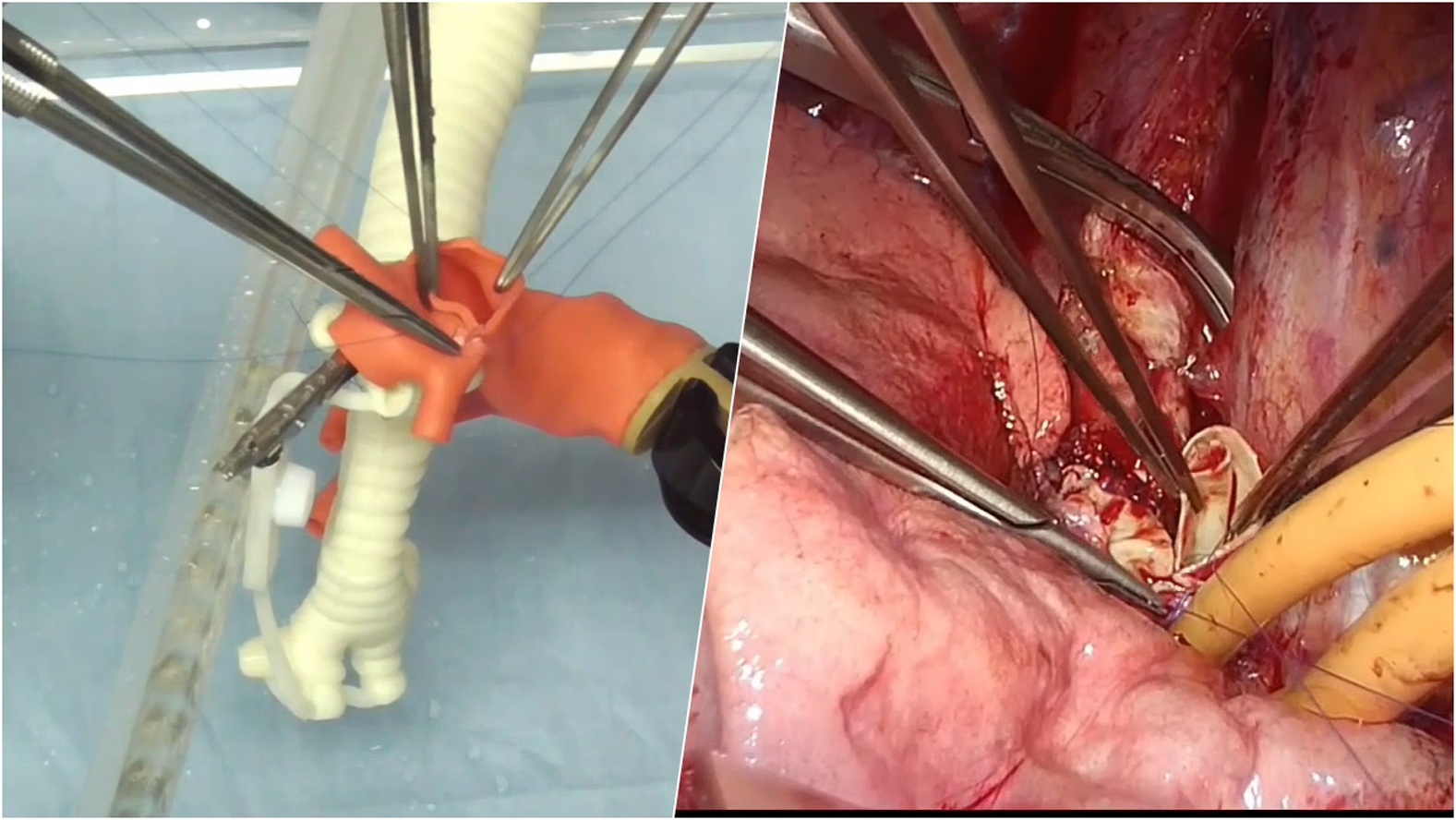

Supplement: Video 1 — Comparison of the right upper double-sleeve resection on the actual patient and on the model performed by the same surgeon. Video available at: https://www.jtcvs.org/article/S2666-2507(24)00446-2/fulltext. [file fx2.jpg]
